# Mechanically Strong, Low Thermal Conductivity and Improved Thermal Stability Polyvinyl Alcohol–Graphene–Nanocellulose Aerogel

**DOI:** 10.3390/gels7040170

**Published:** 2021-10-15

**Authors:** Xiuya Wang, Pengbo Xie, Ke Wan, Yuanyuan Miao, Zhenbo Liu, Xiaojun Li, Chenxi Wang

**Affiliations:** Key Laboratory of Bio-Based Material Science and Technology of Ministry of Education, Northeast Forestry University, Harbin 150040, China; wangxiuya2019@nefu.edu.cn (X.W.); xiepengbo@nefu.edu.cn (P.X.); wkxlqy@nefu.edu.cn (K.W.); miao.yuanyuan@nefu.edu.cn (Y.M.); lixiaojun@nefu.edu.cn (X.L.); wangchenxi@nefu.edu.cn (C.W.)

**Keywords:** ternary composite aerogel, cellulose, graphene, polyvinyl alcohol, graphene aerogel

## Abstract

Porous aerogel materials have advantages of a low density, low thermal conductivity and high porosity, and they have broad application prospects in heat insulation and building energy conservation. However, aerogel materials usually exhibit poor mechanical properties. Single-component aerogels are less likely to possess a good thermal stability and mechanical properties. It is necessary to prepare multiple-composite aerogels by reinforcement to meet practical application needs. In this experiment, a simple preparation method for polyvinyl alcohol (PVA)–graphene (GA)–nanocellulose (CNF) ternary composite aerogels was proposed. This is also the first time to prepare ternary composite aerogels by mixing graphene, nanocellulose and polyvinyl alcohol. A GA–CNF hydrogel was prepared by a one-step hydrothermal method, and soaked in PVA solution for 48 h to obtain a PVA–GA–CNF hydrogel. PVA–GA–CNF aerogels were prepared by freeze drying. The ternary composite aerogel has advantages of excellent mechanical properties, a low thermal conductivity and an improved thermal stability, because strong hydrogen bonds form between the PVA, GA and CNF. The composite aerogels were characterized by scanning electron microscopy, Fourier transform infrared spectroscopy, X-ray diffractometry, Brunauer–Emmett–Teller analysis, dynamic thermal analysis, thermogravimetry and thermal constant analysis to characterize the properties of the ternary composite aerogels. The lightweight, low-density and porous PVA–GA–CNF composite aerogels withstood 628 times their mass. The thermal conductivity of the composite aerogels was 0.044 ± 0.005 W/mK at room temperature and 0.045 ± 0.005 W/mK at 70 °C. This solid, low thermal conductivity and good thermal stability PVA–GA–CNF ternary composite aerogel has potential application in thermal insulation.

## 1. Introduction

Aerogel materials have structural characteristics of a low density, high specific surface area, high porosity and high pore volume [1]. They have special optical, thermal, acoustic and electrical properties, such as a high temperature resistance, low thermal conductivity, low refractive index and low acoustic propagation speed [2,3,4]. Aerogel materials have broad application prospects in many fields, such as thermal insulation, adsorption and separation, biomedicine, photoelectrocatalysis, energy storage conversion, sound absorption and insulation, and high-energy particle capture [5]. These properties have attracted widespread attention in scientific research, production, and design, and have become a key research field in material science [6]. Aerogels perform better than traditional thermal-insulation materials, such as rock wool board, glass wool, polystyrene foam and extruded polystyrene foam board, in the field of heat insulation [7,8]. The average aerogel pore size is ~20 nm, which is much smaller than the average free path of air (70 nm) [9]. Their convective heat transfer is low, which allows for a significant reduction in gas heat conduction.

Graphene (GA) aerogels are also termed GA foams, sponges or macrostructures [10,11,12]. GA aerogels have a porous interconnected three-dimensional sponge-like network structure that is dominated by GA. As a new nanoporous material, GA aerogels have a high hydrophobicity, high specific surface area, high porosity and good chemical stability [13,14,15]. However, because of the existence of van der Waals and π–π bond forces between GA sheets, GA aerogels are prone to irreversible stacking and agglomeration, which diminishes the advantages of the GA. GA aerogels have limited practical application because of their inadequate mechanical properties and their difficulty in combining the multiple performances of single-component aerogels [16]. The introduction of other functional materials into GA aerogels can alleviate GA agglomeration and compensate for defects in the use of individual materials [17,18,19]. The preparation of multiple-composite aerogels with an excellent performance is of high research significance.

As a natural polymer, nanocellulose (CNF) has a good biocompatibility and can be used as a reinforcement unit in composites [20]. When nanocellulose is added into GA as a nanofiller, it interferes with hydrogen bond formation between GA sheets, slows down π–π stacking, and enhances composite mechanical strength [21,22,23]. Zhang et al. prepared composite aerogels with excellent mechanical properties by mixing cellulose nanofibers and GA through atmospheric-pressure drying technology [24]. Cellulose nanofibers are one of the most suitable raw materials for thermally insulated biomass-based organic aerogels. Yue et al. used directional fibers as channel walls, and aligned N-doped GA sheets along the in-plane direction to prepare oriented GA. This aerogel showed a thermal conductivity in the thickness direction (26.6–29.8 mW/mK) that was lower than that in the plane (44.9–55.1 mW/mK) and a thermal conductivity in the plane that was as low as 23.3 mW/mK after heat treatment at 300 °C [25].

In addition to the advantages of GA and CNF use in composite aerogel preparation to yield excellent mechanical and thermal insulation properties, polyvinyl alcohol (PVA) with a high strength, good toughness and low price can also be used. PVA is rich in hydroxyl groups and can form strong hydrogen bonds between GA and CNF to enhance the mechanical properties of the composites [26,27]. This is also the first time to prepare ternary composite aerogels by mixing graphene/nanocellulose and polyvinyl alcohol. In this experiment, a method to prepare PVA–GA–CNF ternary composite aerogel was proposed. By adjusting the content of the three materials, ternary composite aerogels with excellent mechanical properties, a high thermal stability, and a strong heat storage capacity were obtained. The network structure of GA aerogels is porous, and CNF and PVA addition arranges the pores more closely. A high porosity and small pore size can reduce the heat transfer rate, improve the mechanical properties of the composites and enhance the potential application value of PVA–GA–CNF ternary composite aerogels in flexible thermal insulation materials, including civil and commercial buildings, aerospace and commercial aircrafts.

## 2. Results

### 2.1. Microstructure and Chemical Characterization

#### 2.1.1. SEM

**Figure 1:**
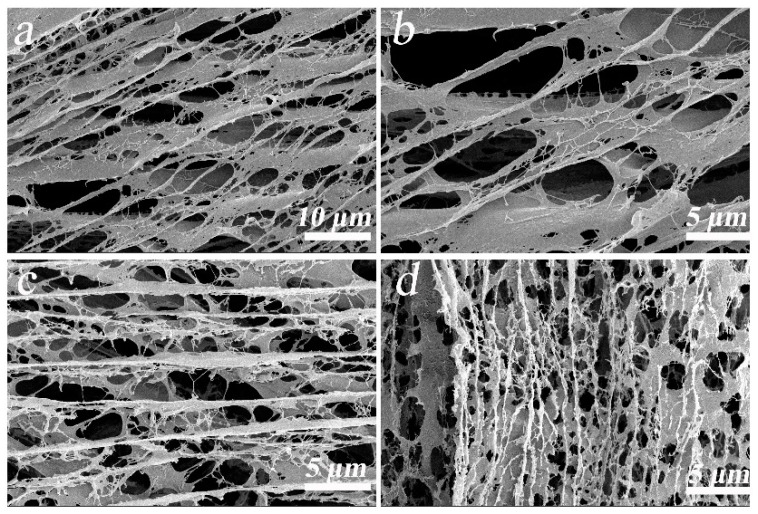
SEM micrographs of (**a**,**b**) PVA–GA–CNF-2 aerogel, (**c**,**d**) PVA–GA–CNF-4 and PVA–GA–CNF-5 aerogels, respectively.

The SEM images in Figure 1 show that the composite aerogel samples had a three-dimensional porous structure. In Figure 1a,b, the filamentous CNF formed a close connection with the GA sheet. Figure 1b–d shows that at the same magnification, an increase in CNF content increases the number of pores in the composite aerogel, and the pore walls thicken, which may be because of the role of CNF in the composite. The porous structure reduces the thermal diffusion of composite aerogels and enhances the thermal storage capacity. The supporting effect of CNF provides a microstructure explanation for the increase in composite aerogel mechanical properties.

#### 2.1.2. FTIR

**Figure 2:**
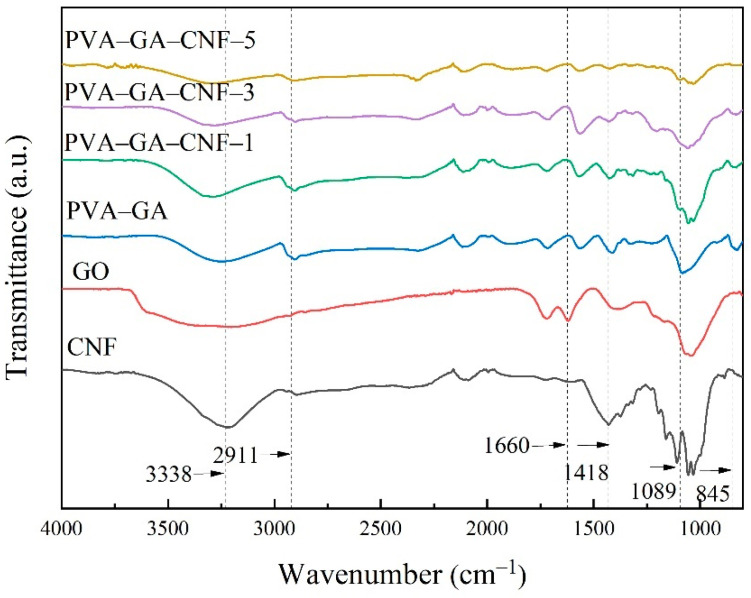
Infrared spectra of GO, CNF, PVA–GA aerogels and PVA–GA–CNF-(1, 3, 5) aerogels.

A comparison of the PVA–GA and GO curves (Figure 2) shows that the symmetric stretching of C–H at 2911 cm^−1^, the stretching of C–O–C at 1089 cm^−1^ and the stretching of C–C at 845 cm^−1^ are the main peaks of PVA, which indicates that PVA has been added to the PVA–GA aerogel, and these peaks are reflected in the PVA–GA–CNF aerogel [28]. A comparison of the PVA–GA and GO curves shows that the peak of −OH at 1660 cm^−1^ in the PVA–GA curve disappeared, which indicates that the GA oxide was reduced to GA, which resulted in a reduction in −OH bond content [29,30,31,32]. The curves of PVA–GA to PVA–GA–CNF-5 show that an increase in CNF moved the peak of O–H at 3200–3500 cm^−1^ to a higher wave number, and the peak of C–H at 1418 cm^−1^ was weakened, which indicates that many O–H and C–H in the composite formed hydrogen bonds. Hydrogen bonds can form between GA, CNF and PVA, which explains the later enhancement in mechanical properties of the composite aerogels.

#### 2.1.3. XRD

**Figure 3:**
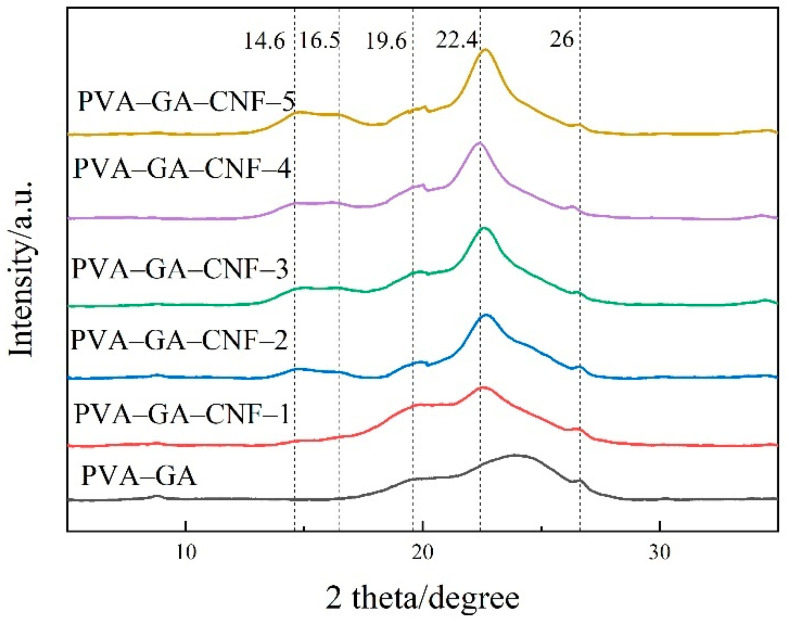
XRD patterns of PVA–GA aerogels and PVA–GA–CNF-(1, 2, 3, 4, 5) aerogels.

Figure 3 shows the XRD patterns of PVA–GA aerogels and PVA–GA–CNF-(1–5) aerogels. Four obvious diffraction peaks were present. The diffraction peak at 22.4° is the characteristic peak of GA, and the peaks at 16.5° and 22.7° correspond to the 10° plane and 002 plane of CNF, respectively [33]. The peak at 9.6° was the orthogonal lattice structure of semi-crystalline PVA [34]. The characteristic peaks of GA oxide did not appear at 11.7° and 11.2° in all samples, which indicates that GA oxide was transformed into GA [35]. These results are consistent with the FTIR test results. GA, CNF and PVA exist simultaneously in PVA–GA–CNF ternary composite aerogels. An increase in CNF content yields a more obvious characteristic CNF peak, and the GA peak shifts to the left. According to the Bragg equation, the lamellar spacing of GA increases because GO removes many oxygen-containing functional groups during reduction, and GA is restacked under the action of a π–π bond. However, CNF can be inserted between GA sheets to slow down the π–π stacking between GA sheets, which explains the enhancement in mechanical properties of the composite aerogels.

#### 2.1.4. BET

**Figure 4:**
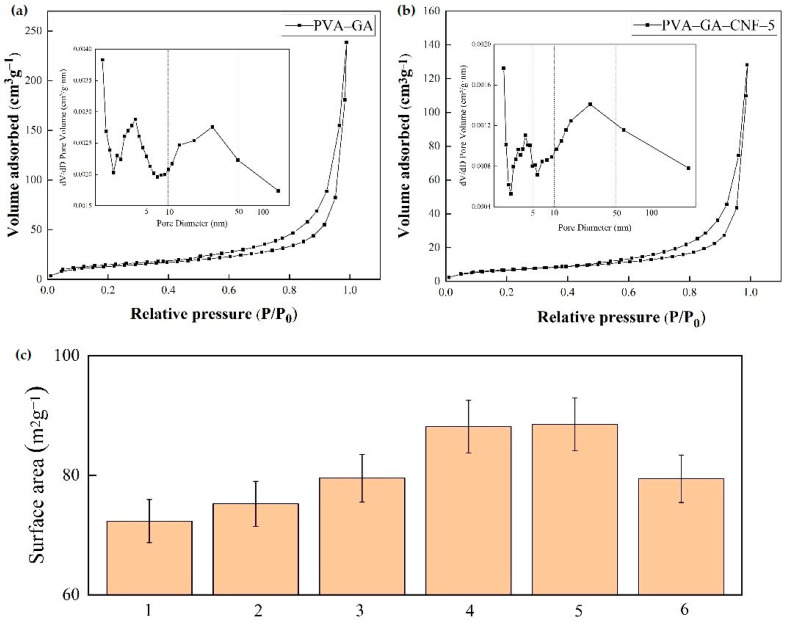
Pore size distribution and specific surface area of composite aerogels. (**a**,**b**) BET diagrams of PVA–GA and PVA–GA–CNF-5 aerogels, respectively. (**c**) Specific surface areas of PVA–GA and PVA–GA–CNF-(1, 2, 3, 4, 5) aerogels.

The obvious type-IV physical adsorption isothermal curve in Figure 4a,b shows that all test samples had a typical mesoporous structure. This is consistent with the dense porous structure of the composite aerogel proved by SEM test. The formation of an H3 hysteresis loop was caused by nitrogen eruption from the gap between the mesopores and the wrinkled wide gap that accumulated in the GA layer during desorption [36]. Figure 4c shows that the PVA–GA and PVA–GA–CNF-(1, 2, 3, 4, 5) aerogels have a high specific surface area. With an increase in CNF content, the specific surface area of the ternary composite aerogels increased initially and then decreased because CNF is a nanofiller with a high specific surface area. The connection between the CNF and GA makes the composite aerogel pore structure denser, and the specific surface area of the composite materials increases gradually [37]. When the CNF content is too high, the composite aerogel structure collapses. When the sample density is too large, the dense connection within the composite reduces the void volume in the aerogel, which reduces the specific surface area of the material slightly. However, the specific surface area of the PVA–GA–CNF-5 aerogel remains higher than that of the PVA–GA aerogel. The dense pore structure of the ternary composite aerogel improves the heat storage and thermal insulation effect of the PVA–GA–CNF ternary composite aerogel.

### 2.2. Mechanical Property

**Figure 5:**
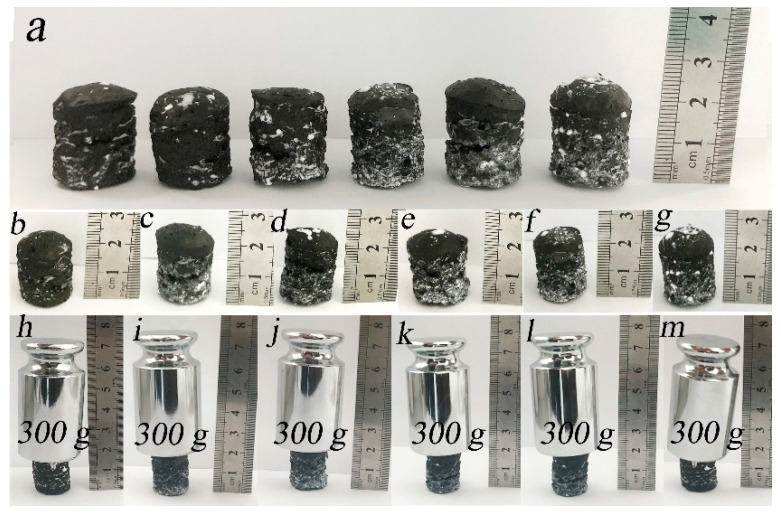
Compression test images of PVA–GA aerogel and PVA–GA–CNF-(1–5) aerogel. (**a**) Appearance of PVA–GA aerogel and PVA–GA–CNF-(1, 2, 3, 4, 5) aerogel. (**b**–**m**) Height changes of PVA–GA aerogel and PVA–GA–CNF-(1–5) aerogel under 300 g mass, respectively.

Figure 5a shows the appearance of the prepared PVA–GA aerogels and PVA–GA–CNF-(1–5) aerogels. All samples were cylinders with a radius of ~0.9 cm and a height of ~2 cm. White flocculent spots appear on the cylinder surface, which is the solid form of polyethylene solution that is attached to the composite hydrogel surface after freeze-drying. When PVA–GA aerogels and PVA–GA–CNF-(1–5) aerogels were subjected to a 300-g (~567 times their own mass) mass, they were not crushed, and their heights changed only slightly. These samples were subjected to a higher mass than the maximum mass (200 g) that the CNF/PVA/GO carbon coatings, as prepared by Xu et al. through freeze drying and carbonization, withstood [38].

**Figure 6:**
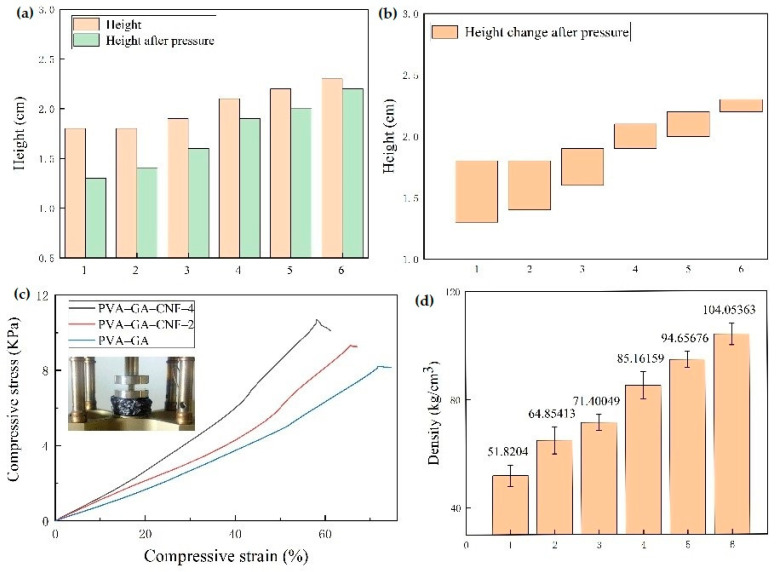
(**a**) Height diagram, (**b**) height change diagram after pressure, (**c**) stress–strain curve and (**d**) density numerical diagram of PVA–GA aerogel and PVA–GA–CNF-(1–5) aerogel.

Due to the porosity of aerogels, there is more demand for improving the mechanical properties of aerogels. As shown in Figure 6a,b, an increase in CNF content resulted in a decrease in composite aerogel height and an increase in mechanical properties for the same pressure. Hydrogen bonds that formed between PVA, GA and CNF strengthened the mechanical properties of the composites, which could be obtained in XRD and FTIR tests. This also shows that although the aerogels are porous, increasing the number of hydrogen bonds in the composite aerogels by regulating the content of CNF, GA and PVA can also improve the mechanical properties of the composite aerogels. Figure 6c shows that under the same pressure, the strain value of PVA–GA–CNF-4 aerogels with more CNF was low, which proves the role of CNF in enhancing the mechanical properties of the composite aerogels. The maximum allowable pressure of PVA–GA–CNF-4 aerogel reached 10.7 kPa, which is equivalent to 628 times the mass of the aerogel itself. Compared with Mi et al.’s cellulose–GA composite aerogel (7.4 kPa), which was prepared by two-way freeze-drying and chemical vapor deposition grafting of long carbon chains, the maximum pressure that was withstood increased by 45%, and the maximum pressure was 78% larger than that of the GA/cellulose nanocrystalline aerogel (6 kPa) as prepared by Zhang et al. through a two-step reduction and atmospheric drying method [24,39]. Therefore, PVA can improve the mechanical properties of the composite aerogels. In Table 1, it can be seen that the maximum allowable pressure of composite aerogels prepared by graphene, nanocellulose and PVA reported in recent years. These values are slightly less than 10.7 kPa of PVA–GA–CNF-4 aerogel. It can be seen that the strong hydrogen bonds formed between graphene, nanocellulose and PVA play a crucial role in enhancing the mechanical properties of composite aerogels. Figure 6d shows that the ternary composite aerogel density increased gradually with an increase in CNF content because all samples had the same volume during the hydrogel preparation, which can be seen in Figure 5. Cylindrical samples with a radius of 0.9 cm and a height of ~2 cm were prepared by using a 25 mL polytetrafluoro-lined hydrothermal synthesis reactor. An increase in CNF content increased the number of hydrogen bonds that formed by the combination of GA and PVA, and the pore structure was denser. The mechanical properties of the composite aerogel, the heat insulation and the thermal insulation effect of the composite aerogel were enhanced.

### 2.3. Thermal Properties

**Figure 7:**
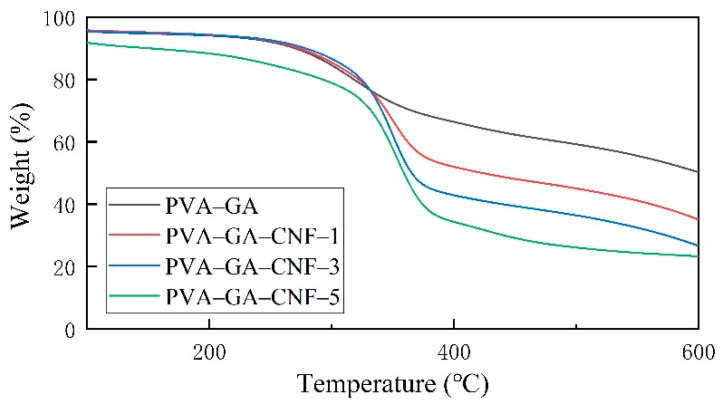
Thermogravimetric analysis of PVA–GA aerogels and PVA–GA–CNF-(1, 3, 5) aerogels.

Figure 7 shows that all samples displayed a significant mass loss step, mass loss temperature range of 350–380 °C, and almost no mass loss before 200 °C. The mass loss was divided into three main stages. The first stage occurred at 0–120 °C, and the thermal mass loss of all samples was within 5%. As proved by SEM and BET tests, the composite aerogels showed porous structure. The mass loss resulted mainly because of the presence of residual water in the aerogel pores, whereas the sample itself displayed no mass loss [50]. Obvious mass loss occurred in the second stage. The temperature range in this stage was 330–360 °C, and the thermal mass loss was 34–55%. The main reasons for the mass loss in this stage were cellulose pyrolysis and PVA–CNF chain decomposition [51]. The third stage occurred at 600–700 °C, and the thermal mass loss was 20–30%. At this stage, the carbon residue was oxidized and degraded to produce low-molecular-mass gas substances. The corresponding thermogravimetric loss of the measured samples was 20 wt%, which corresponded to 318, 321, 324 and 292 °C, respectively, and was higher than those of the PVA–CNF mixed carbon aerogels as prepared by Huang et al. through ultra-low phosphorus freeze drying (243 °C) [52]. An increase in CNF content resulted in an initial increase and then a decrease in thermal stability of the composite aerogels because the tight connection of formed hydrogen bonds increased the thermal stability of the composite materials. However, because of the pyrolysis of a large amount of CNF when the CNF content was high, the thermal stability of the sample with the highest CNF content (PVA–GA–CNF-5) was reduced to a certain extent. In the practical application of composite aerogels in heat insulation and heat preservation, it is important to control the CNF content and maximize the role of CNF.

**Figure 8:**
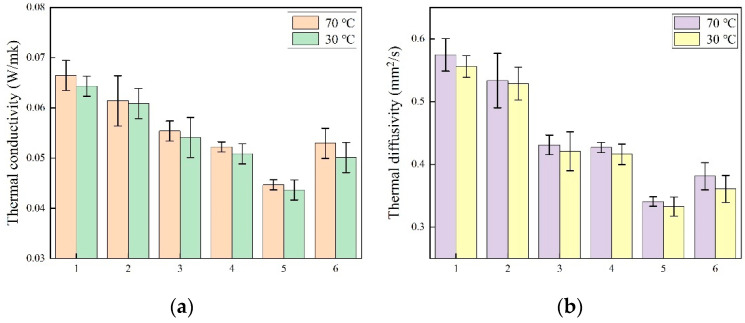
(**a**) Thermal conductivity of PVA–GA aerogels and PVA–GA–CNF-(1–5) aerogels. (**b**) Thermal diffusivity of PVA–GA aerogels and PVA–GA–CNF-(1–5) aerogels.

The thermal conductivity and thermal stability of the PVA–GA aerogel and PVA–GA–CNF ternary composite aerogel are shown in Figure 8. In some insulation and insulation applications, materials require a low thermal conductivity, thermal diffusivity and high thermal stability. As the heat transfer in aerogels is controlled mainly by the gas phase (air), the heat transfer rate is significantly lower than that of the solid phase and/or radiation [53,54]. The heat transfer in aerogels occurs through gas conduction, solid conduction and infrared radiation transfer. The solid conduction increases with an increase of density, whereas the gas conduction and infrared radiation transfer decrease with an increase in density [55].

The thermal conductivity and thermal diffusion coefficient of the PVA–GA aerogel and PVA–GA–CNF ternary composite aerogel at 30 °C and 70 °C were tested. Figure 8 shows that the thermal conductivity of all samples at 70 °C exceeded that at 30 °C, which can be attributed to the solid–liquid phase transition, which is consistent with previous literature [56]. With an increase in CNF content, the thermal conductivity and thermal diffusivity of the composite aerogels decreased initially and then increased because the increase in CNF content increased the aerogel density, the voids of the composite aerogels were more dense, and the number of hydrogen bonds that formed by F between PVA, GA and CN increased. An improved heat storage in the voids yields a smaller loss. However, when the CNF content was too high, the composite aerogel density increased, and the solid conduction ability increased, which increased the thermal conductivity of the composite aerogel slightly. The thermal conductivity of the PVA–GA–CNF-4 aerogel with the smallest thermal conductivity was 0.044 ± 0.005 W/mK at 30 °C and 0.045 ± 0.005 W/mK at 70 °C, which is 35% lower than the thermal conductivity (0.067 W/mK) of the GA aerogels that were prepared by Cheng et al. through hydrothermal reduction and supercritical ethanol drying, and 75% lower than the thermal conductivity (0.174 W/mK) of cellulose aerogels with a similar density prepared by Wang et al. [57,58]. In Table 2, it can be seen that the thermal conductivity of composite aerogels prepared by graphene, nanocellulose and PVA reported in recent years at room temperature. These values are slightly larger than 0.044 ± 0.005 W/mK of PVA–GA–CNF-4 aerogel. By controlling the content of nanocellulose and the number of hydrogen bonds in the composite aerogels, the composite aerogels show a dense porous structure, which plays a significant role in reducing the thermal conductivity of the composite aerogels and enhancing their thermal storage capacity. Ternary porous aerogel structures prepared by the interaction between GA, CNF and PVA reduced the composite thermal conductivity.

The thermal diffusivity was used to evaluate the response speed of composite aerogels to ambient temperature changes, which is related to the heat transfer efficiency. Compared with the thermal diffusivity of composite aerogels at 70 °C, a slight decrease in thermal diffusivity of the molten state at 30 °C occurred because of the increase in specific heat during the solid–liquid phase transformation [59]. The PVA–GA–CNF-4 aerogel had the smallest thermal diffusion coefficient; the thermal diffusion coefficient at 30 °C was 0.333 ± 0.001 mm^2^/s, and the thermal diffusion coefficient at 70 °C was 0.341 ± 0.015 mm^2^/s.

**Table 2:** Thermal conductivity of CNF composite aerogel, GA composite aerogel and PVA composite aerogel.

Composite Aerogels	Thermal Conductivity (W/mK)	Reference
graphene aerogel	0.0667	[57]
cellulose aerogel	0.174	[58]
polyvinyl alcohol-cellulose acetate aerogel	0.049	[60]
tetradecanoyl-graphene aerogel	0.498	[61]
graphene-carbon nanotube aerogel	0.76	[62]
nanocellulose aerogel	0.105	[63]
cellulose nanofibers aerogel	0.12	[64]
polyvinyl alcohol-cellulose nanofibers-gelatin aerogel	0.047	[41]
waste tissue paper-polyvinyl alcohol-carbon aerogel	0.087	[65]
attapulgite-polyvinyl alcohol-cotton cellulose nanowhisker-melamine aerogel	0.045	[66]

## 3. Conclusions

A preparation method for ternary composite aerogels composed of PVA, GA and CNF was proposed. This is also the first time to prepare ternary composite aerogels by mixing graphene/nanocellulose and polyvinyl alcohol. The microstructure and composition of the composite aerogels were determined by various characterization methods, such as SEM, XRD and FTIR. The pore structure and specific surface area of the composite aerogels were determined by BET. Thermogravimetric and thermal constant analysis showed that the thermal conductivity of the PVA–GA–CNF-4 aerogel was smallest at 0.044 ± 0.005 W/mK at 30 °C, the thermal diffusion coefficient was 0.333 ± 0.001 mm^2^/s, the thermal conductivity was 0.045 ± 0.005 W/mK at 70 °C, and the thermal diffusion coefficient was 0.341 ± 0.015 mm^2^/s. The mechanical properties of the PVA–GA–CNF composite aerogels were tested by using the DMA compression test. PVA–GA–CNF-4 aerogels displayed a good compression resistance, and withstood a pressure of 10.7 kPa, which was equivalent to 628 times the mass of the aerogel itself. PVA, GA and CNF were connected by hydrogen bonds, and CNF could be inserted between GA sheets, which slowed down the π–π stacking of GA and enhanced the composite mechanical properties. This porous, lightweight, low-density ternary composite aerogel with a high compression performance, good thermal stability, low thermal conductivity and thermal diffusion coefficient is expected to become an economical, efficient and environmentally friendly substitute for insulation materials in housing, clothing, aerospace and other industries.

## 4. Materials and Methods

### 4.1. Materials and Chemicals

Graphite crystal (99 wt.%) was from Jiangsu Changde High-Tech Carbon Materials Co., Ltd. (Changde, China). Sodium nitrate (NaNO_3_) was from Shanghai Yixin Chemical Co., Ltd. (Shanghai, China). Potassium permanganate powder (KMnO_4_) was from Qufu Xinxin Chemical Co., Ltd. (Qufu, China). Sulfuric acid (H_2_SO_4_ 98 wt.%) and hydrogen peroxide (H_2_O_2_ 30 wt.%) was from Anshan Anji Chemical Co., Ltd. (Anshan, China). Deionized water was from Sigma-Aldrich (Harbin, China). Microcrystalline cellulose powder (MCC, particle size: 50 µm) and polyvinyl alcohol (PVA) were from Shanghai Aladdin Biochemical Technology Co., Ltd. (Shanghai, China).

### 4.2. Graphene Oxide (GO) Preparation

The improved Hummers method was used in which 46 mL of concentrated sulfuric acid was placed in an ice bath and cooled to 4 °C. Under the action of a magnetic stirrer, 2 g of graphite powder, 1 g sodium nitrate and 6 g potassium permanganate were added slowly to the concentrated sulfuric acid. The solution temperature during the reaction was controlled at 5–10 °C and maintained for 90 min. The mixed solution was transferred to a water bath and maintained at 35–40 °C for the intermediate-temperature reaction. The mixed solution was removed 30 min later for the high-temperature reaction in which 92 mL deionized water was added dropwise to the reaction solution. During the reaction, the water addition rate and amount were controlled to keep the reaction solution temperature at ~95 °C for at least 15 min. When the solution changed from dark-green to red–brown, the high-temperature reaction ended. A 30 wt% hydrogen peroxide solution was added to the solution until no bubbles were generated. At this time, the solution turned golden-yellow and then turquoise. The obtained solution was subjected to ultrasonic treatment for 30 min, followed by precipitation, centrifugation and dialysis to obtain a high-purity GA-oxide solution. 

### 4.3. Nanocellulose (CNF) Preparation

According to the Bondeson D method, microcrystalline cellulose was acidified into CNF [27]. Sulfuric acid (63.5%) was prepared for acidolysis MCC. MCC solid was dissolved in deionized water to obtain MCC aqueous solution. The MCC aqueous solution was placed on a magnetic stirrer, and 63.5% sulfuric acid was added to the MCC aqueous solution, with stirring for 2 h until the solution was mixed uniformly. The suspension was centrifuged repeatedly and washed with deionized water (at least five times). After centrifugation, dialysis was performed until the washing water had a constant pH close to neutral. The sample was placed in water that contained ice with ultrasonic treatment for 2 h. Ice was added to keep the temperature below room temperature. The CNF solution was frozen by liquid nitrogen, and placed in a freeze dryer to freeze and dry the solution into a powder to yield solid CNF.

### 4.4. PVA–GA–CNF Aerogel Preparation

**Figure 9:**
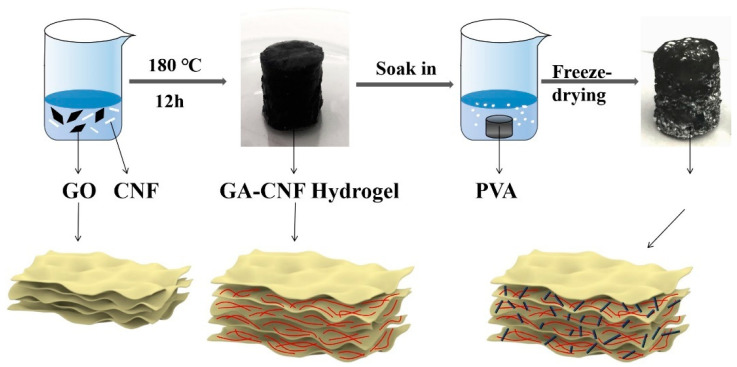
Preparation process of PVA-GA-CNF aerogels.

The preparation process is shown in Figure 9. PVA (12.5 g) was dissolved in 50 mL deionized water and stirred at 80–100 °C for 2 h to prepare a 20 wt% PVA solution. Five GO powders (250 mg) were weighed and dissolved in 10 mL deionized water. CNF powder (0, 50, 100, 150, 200, and 250 mg) was added with stirring for 3 h to prepare a GO solution and GO–CNF mixed solution. Six groups of samples were placed in a 25 mL polytetrafluoroethylene (PTFE) lined hydrothermal reactor. The hydrothermal reactor was placed in an oven and heated at 180 °C for 12 h. After heating, the hydrothermal reactor was cooled to room temperature, and GA hydrogel and GA–CNF hydrogel were obtained. PVA–GA hydrogel and PVA–GO–CNF hydrogel were prepared by immersing six groups of hydrogels in 20 wt% PVA solution for 48 h. All hydrogel samples were frozen in liquid nitrogen and then put into the pre-cooled freeze dryer. After freeze-drying for 48 h, PVA–GA aerogel and PVA–GO–CNF aerogel were obtained and termed PVA–GA, PVA–GA–CNF-1, PVA–GA–CNF-2, PVA–GA–CNF-3, PVA–GA–CNF-4 and PVA–GA–CNF-5 for the 0 mg, 50 mg, 100 mg, 150 mg, 200 mg and 250 mg CNF additions, respectively. All samples are prepared by strict control variable method to ensure that the properties of the samples are not affected by other conditions as far as possible.

### 4.5. Characterization

The quality of aerogels was measured using the Metler electronic balance produced in Switzerland. The samples were characterized by QUANTA200 scanning electron microscope (SEM) produced by FEI, the Netherlands. Fourier transform infrared spectroscopy (FTIR) was performed on Bruker Vertex 70, Germany., and the scanning range was 550–4000 cm^−1^. X-ray diffraction (XRD) analysis was performed by using XRD-6100 from Shimadzu, Japan, at a scanning rate of 5 °C / min from 5 ° to 60 °. BET surface area and pore structure of aerogels were measured by nitrogen adsorption using ASAP2460 Brenner-Emmet-Teller (BET) from Mac Instruments, USA. The sample was cut into a 0.5-cm-high cylinder and compression tests were carried out at room temperature. Dynamic thermal analysis (DMA) using DMA242E Artemis from NETZSCH, Germany. The thermal conductivity and thermal diffusivity of the samples were measured by TPS 2500 S produced by Swedish Hot Disk. The thermogravimetric analysis chart of 5~790 °C was obtained by using high precision thermogravimetric analyzer (TG209F1) of Niche, Germany.

## Figures and Tables

**Table 1:** Maximum pressure of CNF composite aerogel, GA composite aerogel and PVA composite aerogel.

Composite Aerogels	Maximum Allowable Pressure (kPa)	Reference
cellulose-graphene aerogel	7.4	[39]
Graphemecellulose nanocrystalline aerogel	6	[24]
cellulose-graphene aerogel	7.2	[40]
polyvinyl alcohol–cellulose aerogel	9.7	[41]
polyvinyl alcohol-cellulose nanofibrils-graphene oxide hybrid aerogel	4	[42]
Fe_3_O_4_-cellulose-polyvinyl alcohol hybride aerogel	6.5	[43]
NiO-Fe_2_O_3_-reduced graphene oxide-polyvinyl alcohol aerogel	9	[44]
N-doped- reduced graphene oxide aerogel	9.3	[45]
cellulose nanofibrils-graphene nanosheets aerogel	8.9	[46]
polyvinyl alcohol-cellulose nanofibril hybrid aerogel	7.2	[47]
CaCO_3_-decorated cellulose aerogel	4.5	[48]
carbon-cellulose aerogel	5.2	[49]

## Data Availability

The data that support the findings of this study are available from the corresponding author upon reasonable request.

## Sample Availability

Samples of the compounds (PVA–GA aerogel, PVA–GA–NCF-1 aerogel, PVA–GA–CNF-2 aerogel, PVA–GA–CNF-3 aerogel, PVA–GA–CNF-4 aerogel, and PVA–GA–CNF-5 aerogel) are available from the authors.
